# Supplementary material for: Adverse Events of Radioligand Therapy in Patients with Progressive Neuroendocrine Neoplasms: The Biggest Eastern European Prospective Study
Source: Cancers (Basel). 2024 Oct 17;16(20):3509. doi: 10.3390/cancers16203509 (PMC11505743; doi:10.3390/cancers16203509)
Supplement: Supplementary file 1 [file cancers-16-03509-s001.zip › cancers-3265823-supplementary.pdf]

Supplementary Materials: Adverse Events of Radioligand Therapy in Patients with Progressive Neuroendocrine Neoplasms—the Biggest Eastern-European Prospective Study

Adam Daniel Durma, Marek Saracyn, Maciej Kołodziej, Katarzyna Jóźwik-Plebanek, Dorota Brodowska-Kania, Beata Dmochowska, Adrianna Mróz, Beata Kos-Kudła and Grzegorz Kamiński

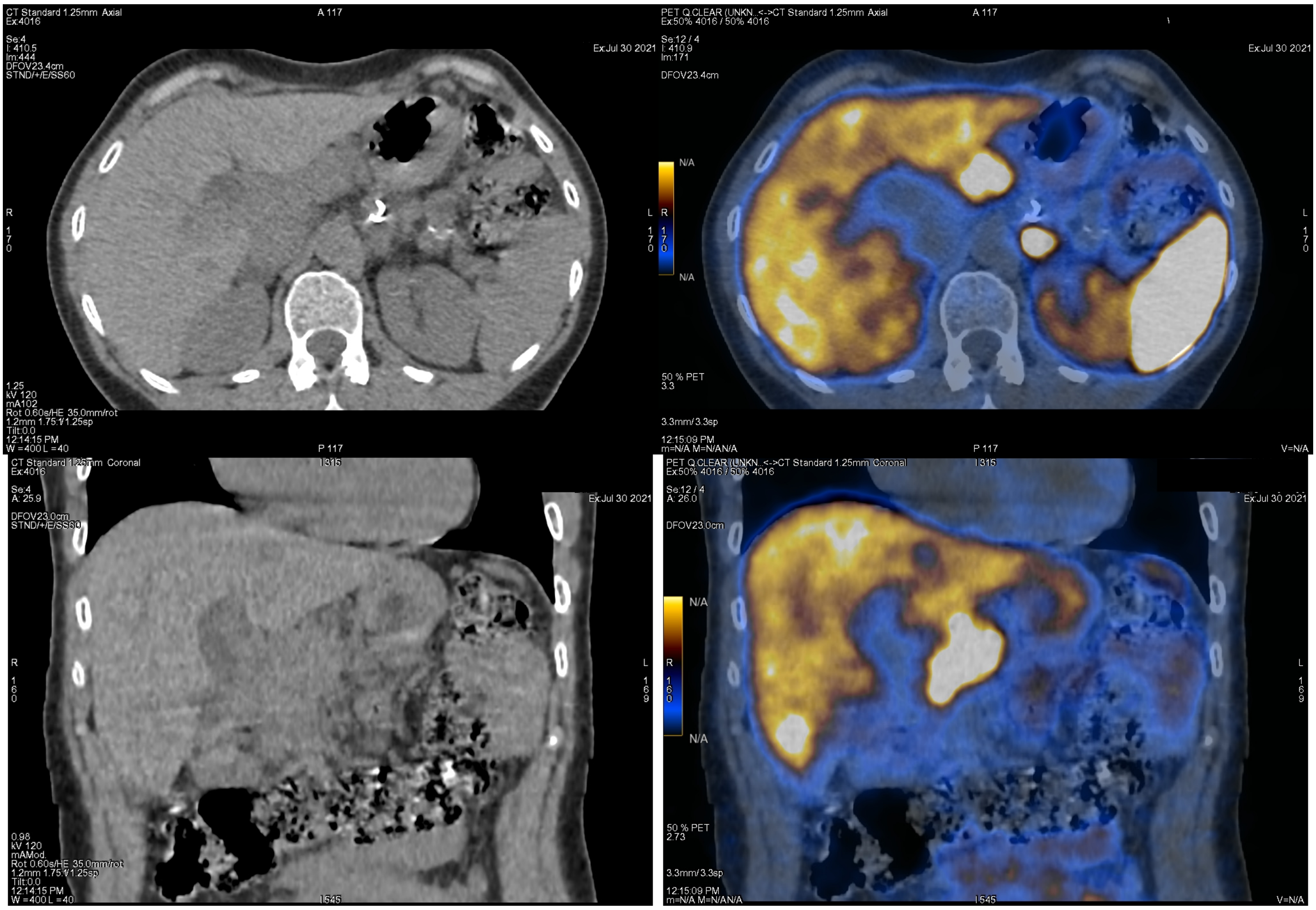

Figure S1. Example of functional study performed during qualification to the study group. PET/CT with [68Ga]Ga-DOTA-TATE (Female, 67y.o.). Visible accumulation of radiotracer in pancreas.

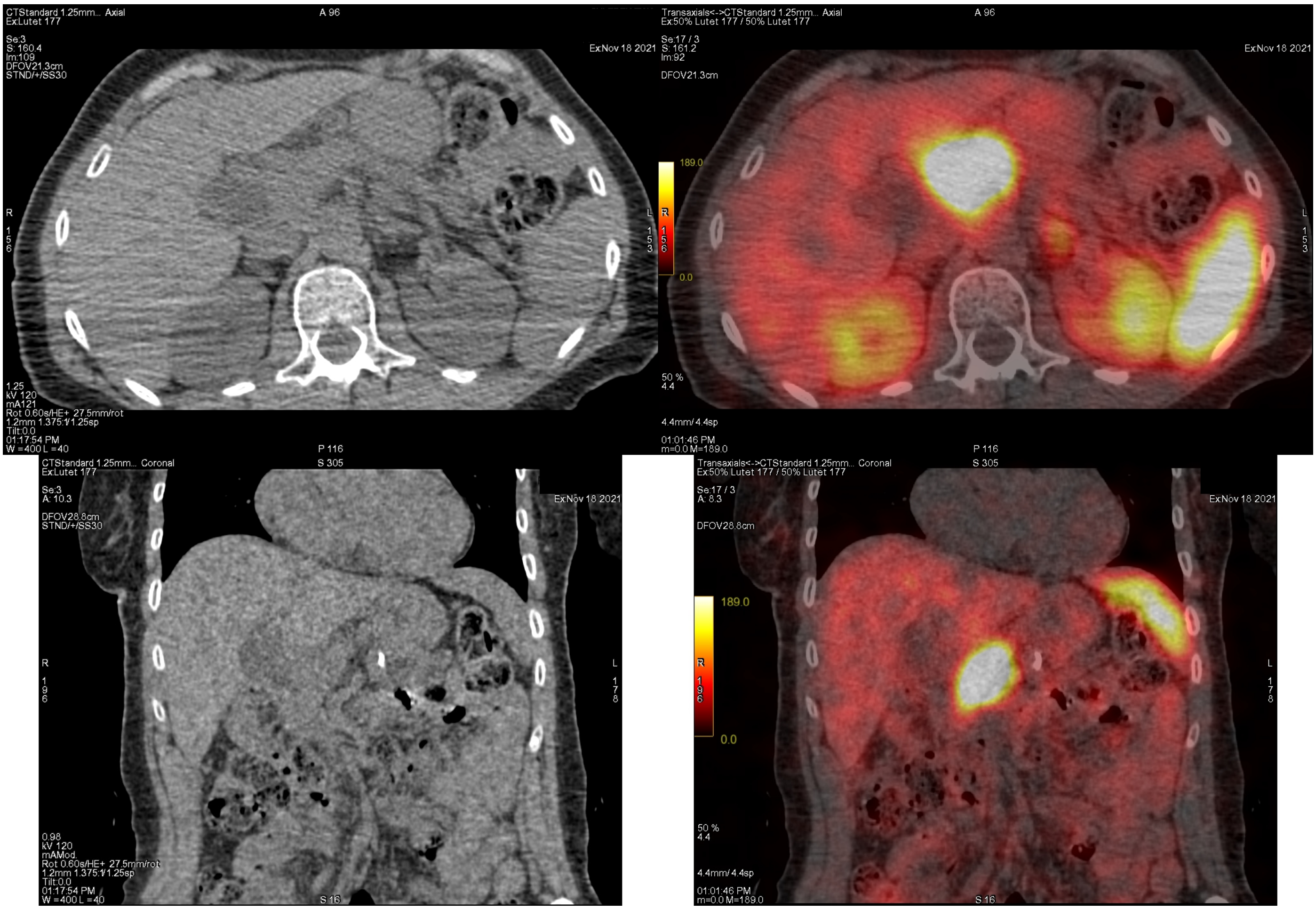

Figure S2. SPECT/CT study (Female, 67y.o.) after Course I of RLT. Visible accumulation of radiotracer in pancreas matching the previous [68Ga]Ga-DOTA-TATE PET/CT study.

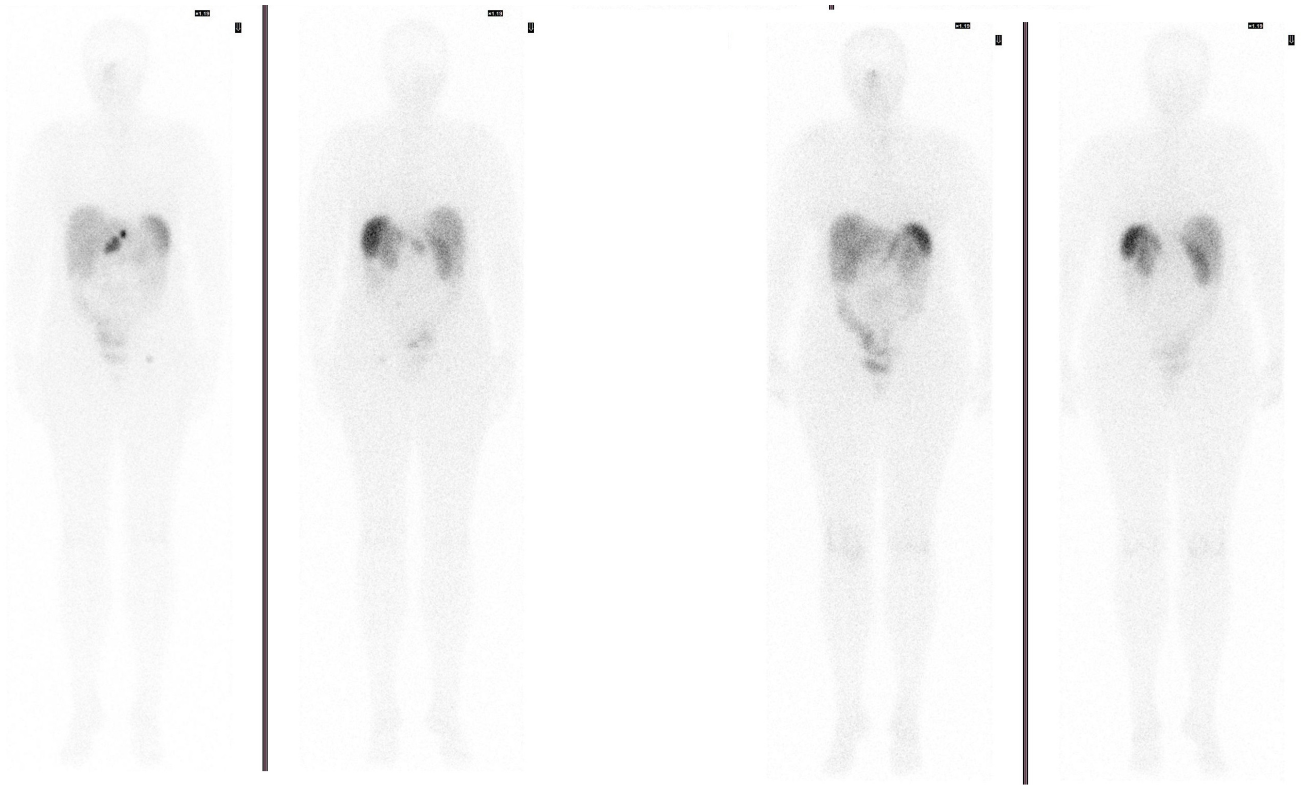

Figure S3. Post-therapeutic planar scintigraphy. Course I vs. Course IV of RLT. (Female, 67y.o.). Visible focal accumulation of radiotracer in pancreas (primary tumor) and in liver (metastasis) after Course I (left scan). Lesions are not visible after Course IV confirming complete regression (CR) (right scan).

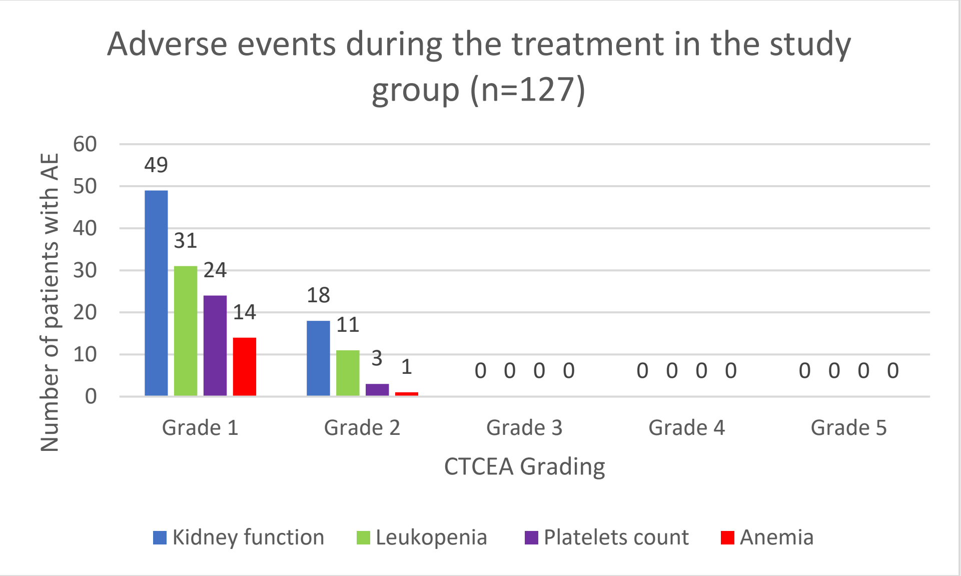

Figure S4. Adverse events during treatment (Course I to Course IV) in the study group (n=127).

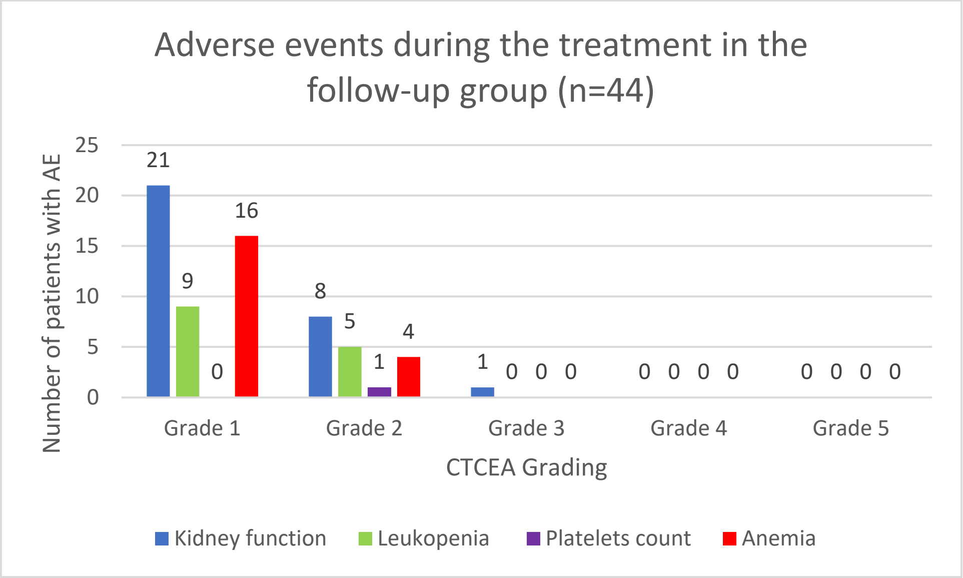

Figure S5. Adverse events in long-term observation of the follow-up group (n=44).

Table S1. Inclusion and exclusion criteria for the study group.

**Inclusion criteria**	**Exclusion Criteria**
Well- and moderately-differentiated unresectable metastatic progressive neuroendocrine neoplasm (defined as Ki-67 < 20%, progression according to the RECIST 1.1 criteria, over the previous 12 months	Lack of consent
Good expression of somatostatin receptors in somatostatin receptor scintigraphy (SPECT/CT) - radiotracer uptake in the majority of the lesions higher than in normal liver (Krenning scale 3) or in [^{68}Ga]Ga-DOTA-TATE PET/CT (SUVmax in the majority of the lesions higher than SUVmax in normal liver);	no tracer uptake in Somatostatin Receptor Imaging,
No possibility of surgical treatment	Karnofsk'y scale <60, WHO/ECOG 3 or 4,
Progression during treatment with long-acting somatostatin analogues	Pregnancy or lactation
	Bone marrow impairment (hemoglobin <8 g/L, or platelets <80.000/µL, or leukocytes <2000/µL, or lymphocytes <500/µL, or neutrophils <1000/µL),
	Renal disfunction - GFR <30 mL/min, or serum Creatinine >1.8 mg/dL
	liver diseases ALT or Bilirubin 3× over upper limit
